# Complete Genome Sequences of *emm111* Type *Streptococcus pyogenes* Strain GUR, with Antitumor Activity, and Its Derivative Strain GURSA1 with an Inactivated *emm* Gene

**DOI:** 10.1128/genomeA.00939-17

**Published:** 2017-09-21

**Authors:** Maria A. Suvorova, Anna N. Tsapieva, Emilie Glad Bak, Valery A. Chereshnev, Ekaterina P. Kiseleva, Alexander N. Suvorov, Manimozhiyan Arumugam

**Affiliations:** a Department of Molecular Microbiology, Institute of Experimental Medicine, Saint Petersburg, Russian Federation; b The Novo Nordisk Foundation Center for Basic Metabolic Research, Faculty of Health and Medical Sciences, University of Copenhagen, Copenhagen, Denmark; c Institute of Immunology and Physiology, Russian Academy of Sciences, Ural Branch, Ekaterinburg, Russian Federation; d Saint Petersburg State University, Saint Petersburg, Russian Federation

## Abstract

We present here the complete genome sequence of *Streptococcus pyogenes* type *emm111* strain GUR, a throat isolate from a scarlet fever patient, which has been used to treat cancer patients in the former Soviet Union. We also present the complete genome sequence of its derivative strain GURSA1 with an inactivated *emm* gene.

## GENOME ANNOUNCEMENT

*Streptococcus pyogenes* is a Gram-positive pathogenic bacterium causing a wide variety of human diseases (1). It has also been used for bacteriolytic cancer therapy starting with “Coley’s toxins” (2) and followed by OK-432 (picibanil) (3), but the mechanisms behind its antitumor activity are unknown. *S. pyogenes* strain GUR, isolated from a scarlet fever patient in the former Soviet Union in 1938, has been used clinically for anticancer treatment in the former Soviet Union for more than 20 years (4) and showed therapeutic effects in several murine cancer models (M. Suvorova, E. P. Kiseleva, and A. N. Suvorov, unpublished data). To identify the role of the antiphagocytic M protein in the cytotoxic activity of this strain, we generated a mutant (strain GURSA1) by inactivating the *emm* gene by insertional mutagenesis (5). Unexpectedly, strain GURSA1 showed better cytotoxic activity in both *in vitro* and *in vivo* experiments on murine malignant tumor cells (M. Suvorova, E. P. Kiseleva, and A. N. Suvorov, unpublished data). To understand the mechanisms of how strains GUR and GURSA1 affect tumor cells, we sequenced their genomes.

Genomic DNA was extracted from overnight cultures of *S. pyogenes* strains GUR and GURSA1 using the MagAttract pathogen kit (Qiagen, USA). Fragment libraries were prepared using the Nextera XT DNA library preparation kit (Illumina, San Diego, CA), followed by 251-bp paired-end sequencing on a MiSeq instrument (Illumina), which This generated 496 Mb and 234 Mb of high-quality paired-end sequence data for strains GUR and GURSA1, respectively.

We first assembled the genome of strain GURSA1 using a custom-built SPAdes assembler version 3.10.0 (6), with the “-k 55,77,99,121,143,151,155” option to specify iterative k-mers. For scaffolding, we used full chromosome sequences from three closely related *S. pyogenes* strains (ATCC 19615, AP1, and AP53, with GenBank accession numbers NZ_CP008926, NZ_CP007537, and NZ_CP013672, respectively) using the “--untrusted-contigs” option. The resulting single scaffold was improved using Pilon version 1.22 software (7). We circularized this gapless chromosome sequence and rearranged it to start at the *dnaA* gene using the Circlator software (8). We then assembled the genome of strain GUR using an identical procedure, with the following modifications: different k-mers with “-k 33,55,77,99,121,143” and providing the chromosome sequence of GURSA1 with the “--untrusted-contigs” option. The resulting single scaffold was also improved, circularized, and rearranged. The genomes of strains GUR and GURSA1 consisted of 1,890,204 bp and 1,893,171 bp, respectively. They encoded 1,820 and 1,825 proteins, respectively, predicted by NCBI’s Prokaryotic Genome Annotation Pipeline (9). Both genomes had 38.5% G+C content, harbored 67 tRNA genes, and encoded 6 copies of the rRNA operon. Using the PHASTER online tool (10), we identified five intact prophage regions with high homology to prophage regions from M3 serotype *S. pyogenes* strain MGAS315. Other than the genomic changes introduced during mutagenesis, GUR and GURSA1 had four single-nucleotide changes between them. Three of these changes were synonymous mutations within the third prophage region, and the fourth change was a nonsynonymous (A284V) change in the Hpr kinase/phosphorylase gene. We assigned type *emm111* to strain GUR using the instructions provided by the Centers for Disease Control and Prevention’s *Streptococcus* Laboratory (https://www.cdc.gov/streplab/).

### Accession number(s).

The genome sequences of strains GUR and GURSA1 were deposited in GenBank under the accession numbers CP022354 and CP022206, respectively. The versions described in this paper are the first versions.
